# Comparative evidence for a link between Peyer's patch development and susceptibility to transmissible spongiform encephalopathies

**DOI:** 10.1186/1471-2334-6-5

**Published:** 2006-01-11

**Authors:** Suzanne G St Rose, Nora Hunter, Louise Matthews, James D Foster, Margo E Chase-Topping, Loeske EB Kruuk, Darren J Shaw, Susan M Rhind, Robert G Will, Mark EJ Woolhouse

**Affiliations:** 1 Centre for Infectious Diseases, University of Edinburgh, Ashworth Laboratories, King's Buildings, West Mains Road, Edinburgh EH9 3JF, UK; 2 Institute for Animal Health, Neuropathogenesis Unit, West Mains Road, Edinburgh EH9 3JF, UK; 3 Institute of Biomedical and Life Sciences, University of Glasgow, Glasgow G12 8QQ, UK; 4 Institute of Evolutionary Biology, School of Biological Sciences, University of Edinburgh, Edinburgh EH9 3JT, UK; 5 Division of Veterinary Clinical Studies, Royal (Dick) School of Veterinary Studies, University of Edinburgh, Easter Bush Veterinary Centre, Roslin, Midlothian EH25 9RG, UK; 6 Division of Animal Health and Welfare, Royal (Dick) School of Veterinary Studies, University of Edinburgh, Easter Bush, Roslin, Midlothian EH25 9RG, UK; 7 The National Creutzfeldt-Jakob disease Surveillance Unit, Western General Hospital, Crewe Road, Edinburgh EH4 2XU, UK

## Abstract

**Background:**

Epidemiological analyses indicate that the age distribution of natural cases of transmissible spongiform encephalopathies (TSEs) reflect age-related risk of infection, however, the underlying mechanisms remain poorly understood. Using a comparative approach, we tested the hypothesis that, there is a significant correlation between risk of infection for scrapie, bovine spongiform encephalopathy (BSE) and variant CJD (vCJD), and the development of lymphoid tissue in the gut.

**Methods:**

Using anatomical data and estimates of risk of infection in mathematical models (which included results from previously published studies) for sheep, cattle and humans, we calculated the Spearman's rank correlation coefficient, r_s_, between available measures of Peyer's patch (PP) development and the estimated risk of infection for an individual of the corresponding age.

**Results:**

There was a significant correlation between the measures of PP development and the estimated risk of TSE infection; the two age-related distributions peaked in the same age groups. This result was obtained for each of the three host species: for sheep, surface area of ileal PP tissue vs risk of infection, r_s _= 0.913 (*n *= 19, P < 0.001), and lymphoid follicle density vs risk of infection, r_s _= 0.933 (*n *= 19, P < 0.001); for cattle, weight of PP tissue vs risk of infection, r_s _= 0.693 (*n *= 94, P < 0.001); and for humans, number of PPs vs risk of infection, r_s _= 0.384 (*n *= 46, P = 0.008). In addition, when changes in exposure associated with BSE-contaminated meat were accounted for, the two age-related patterns for humans remained concordant: r_s _= 0.360 (*n *= 46, P = 0.014).

**Conclusion:**

Our findings suggest that, for sheep, cattle and humans alike there is an association between PP development (or a correlate of PP development) and susceptibility to natural TSE infection. This association may explain changes in susceptibility with host age, and differences in the age-susceptibility relationship between host species.

## Background

The incidence of natural cases of transmissible spongiform encephalopathies (TSEs) or prion diseases is related to age: scrapie incidence in sheep typically peaks between 2 and 3 years of age [1], bovine spongiform encephalopathy (BSE) incidence in cattle peaks at around 5 to 7 years of age [2] and variant Creutzfeldt-Jakob disease (vCJD) incidence in humans peaks at 25 to 30 years [3]. Age-related patterns in incidence will reflect the incubation period of the disease (typically long relative to host life expectancy), the magnitude of the risk of infection and any age dependency in the risk of infection. Analyses of epidemiological data for scrapie [4], BSE [5] and vCJD [6] have suggested that there is significant age dependency in the risk of infection for all these TSEs. Available evidence suggests that these patterns cannot be fully accounted for by changes in exposure, in which case changes in susceptibility must also play a role. However, to date, there has been no indication of why susceptibility might change with age.

Age dependency in the risk of infection by TSEs will reflect any age dependency in exposure to infection and/or in susceptibility to infection for a given level of exposure. Both of these are likely to be linked to the route of transmission. Although other transmission routes may exist (see below), oral exposure appears to be the most important route of transmission for natural TSE infections in sheep, cattle, deer and mink and for vCJD and kuru in humans [1,3,7-9]. There is evidence for the involvement of Peyer's patches (PPs), part of the gut-associated lymphoid tissue (GALT), in orally transmitted TSE infection. Experimental studies in cattle have demonstrated staining for PrP^Sc ^(the abnormal prion protein) in PP follicles in the distal ileum throughout much of the course of the disease following oral exposure to the BSE agent [7]. In sheep, oral infection with scrapie is thought to occur mainly via the ileal PP, followed by replication in GALT [8]. In mule deer fawns, lymphoid follicles of PPs have been shown to accumulate PrP^Sc ^within a few weeks following oral exposure to chronic wasting disease (CWD) [9]. After oral infection of nonhuman primates with BSE-infected material, PrP^Sc ^is initially detected in PPs [10]. In experimental infections, mice deficient in both tumour necrosis factor and lymphotoxin or in lymphocytes, in which PPs are decreased in number, are highly resistant to oral challenge and their intestines are virtually devoid of infectivity at all times post-challenge [11]. These facts collectively suggest a key role for PPs in the infection dynamics of a range of TSEs.

Early presence of PrP^Sc ^in mouse PPs after oral exposure to scrapie [12] has indicated these structures as being the most probable sites for the intestinal uptake of the TSE agent. Various cell types present in this lymphoid tissue have been implicated as important elements in the uptake and propagation of the infectious agent. PrP^Sc ^staining in the follicular dendritic cells of patients with vCJD [13] and of sheep naturally infected with scrapie [14], as well as staining associated with the luminal border of cells in the follicle-associated epithelium (FAE) of sheep suggest uptake of the TSE agent from the intestinal lumen to the underlying lymphoid tissue [8]. Although important functional differences exist between PP in sheep ileum and those in the duodenum and jejunum, the FAE overlying jejunal and ileal PPs has an efficient mechanism for the transcytosis of luminal material [15,16], including prion proteins, to the underlying lymphoid tissue.

The development of GALT is known to be related to age. In young sheep, cattle and humans, ileal PPs are the major component of GALT possessing an extensive bed of follicular dendritic cells and follicle-associated epithelium. The involution of ileal PPs occurs at around puberty in sheep, cattle and humans [17-19]. However, the age-related changes in PP development are not identical across these three species, providing an opportunity for a comparative study. Our hypothesis is that although the relationships between PP development and age and between susceptibility to TSE infection and age differ in sheep, cattle and humans, there should still be a correlation between PP development and susceptibility for each species.

## Methods

### Anatomical studies

Specimens of ileum were collected from 19 sheep of different ages (0–1 year, 1–2 years and >2 years) from a flock of Cheviot sheep maintained by the Institute of Animal Health Neuropathogenesis Unit (NPU) [20]. The study was limited to animals with no clinical or pathological evidence of intestinal disease. Specimens were obtained from sheep that were either euthanized because of severe arthritis in one or more limbs, died shortly after birth or were culled for flock management reasons. The specimens were opened along their mesenteric borders, and rinsed in cold water. PP tissue and lymphoid follicles were visualised by immersing the intestines in 2% acetic acid for 24 hours, and the follicular content of the patches enhanced by staining with 0.5% methylene blue for 2–5 minutes. PP tissue and lymphoid follicles were easily visualised using this technique.

The terminal ileum (distal 0.6 m of the ileum) was transilluminated on a horizontal X-ray view box and digital images were obtained. Image analysis software (Image-Pro Plus^®^) was then used to calculate the areas of intestine and of PP tissue. The area of PP tissue was recorded as a percentage of the total area of intestinal tissue.

To determine the number of lymphoid follicles, the stained intestine was placed between two glass slides, the upper of which was etched in square centimetres. Individual lymphoid follicles appeared as bright blue spots against a faintly blue background when viewed on the X-ray box. The number of lymphoid follicles in 6 different sections along the length of the terminal ileum was counted by naked eye, starting at 5 cm from its caudal end and selecting 4 cm^2 ^sections at every 10 cm thereon, proximally. Results were recorded as the average number of lymphoid follicles per cm^2 ^of ileum.

Our results are described in terms of area of PP tissue and lymphoid follicle density in the sheep ileum; analyses indicate that these two measures are closely correlated (r_s _= 0.958, *n *= 19, P < 0.001). PP data for cattle and humans were obtained from earlier studies [18,19]. The studies used different measures to quantify PP tissue from those we obtained here for sheep. The cattle data [18] refer to weight of PP tissue in the small intestine of 94 German beef cattle. The human data [19] refer to number of PPs in the normal small intestine of 46 individuals between 15 and 96 years of age. The study was limited to necropsies performed within a few hours of death, and to patients with no clinical history or pathological evidence of gastrointestinal tract disease. A second, smaller study of human PPs indicates that, in humans, number of PPs and area of PP tissue in the distal ileum were correlated across age classes (r = 0.415, *n *= 55, P < 0.01) [21]. As far as we are aware, there are no other quantitative data on PP development with respect to age available for these species but, where direct comparisons are possible, it appears that the different measures reflect the same underlying relationship with age.

### Scrapie incidence data

The NPU Cheviot flock, a closed flock maintained explicitly as a source of natural scrapie infections, has been comprehensively documented and demographic information and epidemiological data on all sheep are available [20]. In this study, analyses were based on data obtained from an outbreak of scrapie, which spanned the years 1985 to 1994 affecting cohorts born between 1983 and 1992. This represents a total of 1,473 sheep of which 34 developed clinical scrapie. In this flock, scrapie occurs in two PrP genotypes, VRQ/VRQ and VRQ/ARQ [20]. (There is no evidence that PrP genotype influences PP development). Further details of the outbreak are given elsewhere [20].

### Age-susceptibility functions

The method for calculating the age susceptibility function for sheep follows that of Boëlle et al. [6] used to derive the age risk function for vCJD. The occurrence of cases in genotype *G *sheep is modelled by a Poisson process in the (age, time) plane with intensity π_*G*_(*a*,*t*) given by:

πG(a,t)=βGrGS(a)∫0aexp⁡[−∫0a′λ(u,t−a+u)du]λ(a′,t−a+a′)hG(a−a′)da′
MathType@MTEF@5@5@+=feaafiart1ev1aaatCvAUfKttLearuWrP9MDH5MBPbIqV92AaeXatLxBI9gBaebbnrfifHhDYfgasaacH8akY=wiFfYdH8Gipec8Eeeu0xXdbba9frFj0=OqFfea0dXdd9vqai=hGuQ8kuc9pgc9s8qqaq=dirpe0xb9q8qiLsFr0=vr0=vr0dc8meaabaqaciaacaGaaeqabaqabeGadaaakeaacqaHapaCdaWgaaWcbaGaem4raCeabeaakiabcIcaOiabdggaHjabcYcaSiabdsha0jabcMcaPiabg2da9iabek7aInaaBaaaleaacqWGhbWraeqaaOGaemOCai3aaSbaaSqaaiabdEeahbqabaGccqWGtbWucqGGOaakcqWGHbqycqGGPaqkdaWdXaqaaiGbcwgaLjabcIha4jabcchaWbWcbaGaeGimaadabaGaemyyaeganiabgUIiYdGcdaWadaqaaiabgkHiTmaapehabaGaeq4UdWgaleaacqaIWaamaeaacuWGHbqygaqbaaqdcqGHRiI8aOGaeiikaGIaemyDauNaeiilaWIaemiDaqNaeyOeI0IaemyyaeMaey4kaSIaemyDauNaeiykaKIaemizaqMaemyDauhacaGLBbGaayzxaaGaeq4UdWMaeiikaGIafmyyaeMbauaacqGGSaalcqWG0baDcqGHsislcqWGHbqycqGHRaWkcuWGHbqygaqbaiabcMcaPiabdIgaOnaaBaaaleaacqWGhbWraeqaaOGaeiikaGIaemyyaeMaeyOeI0IafmyyaeMbauaacqGGPaqkcqWGKbazcuWGHbqygaqbaaaa@749E@

where β_*G *_is the birth rate and *r*_*G *_is the relative susceptibility of genotype *G *individuals, *S*(*a*) is the probability of survival (in the absence of scrapie) until age *a*, *h*_*G *_is the probability density function for the incubation period for genotype *G *individuals, and λ(*a*,*t*) is the per capita rate of infection for individuals of age *a *at time *t*. The expression sums the contribution to the incidence of infection at age *a *and time *t *from animals infected when at age *a*', taking into account the fact that the number of animals available at age *a*' to become infected is reduced by those already infected at age *u*. The low incidence of scrapie in this flock [22] permits modelling of the age and timing of cases as a Poisson process because the course of the outbreak does not significantly impact on the demography of the susceptible sheep.

The survivorship function *S*(*a*) is a Weibull function with mean age of death of 2.99 years [23]. The incubation period distribution is a gamma distribution with a mean of 1.9 years [23]. The birth rate β_*G *_is selected to give the average numbers of sheep of different genotypes born per year. The per capita rate of infection, λ(*a*,*t*) has two parts: a time dependent component *g*(*t*) which is assumed here to be proportional to an exponential function fitted to the incidence of infection; and an age-dependent component *f*(a) which represents the relative susceptibilities of different age classes:

f(a)={f1for0≤a<1f2for1≤a<2f3for2≤a<9
 MathType@MTEF@5@5@+=feaafiart1ev1aaatCvAUfKttLearuWrP9MDH5MBPbIqV92AaeXatLxBI9gBaebbnrfifHhDYfgasaacH8akY=wiFfYdH8Gipec8Eeeu0xXdbba9frFj0=OqFfea0dXdd9vqai=hGuQ8kuc9pgc9s8qqaq=dirpe0xb9q8qiLsFr0=vr0=vr0dc8meaabaqaciaacaGaaeqabaqabeGadaaakeaacqWGMbGzcqGGOaakcqWGHbqycqGGPaqkcqGH9aqpdaGabaqaauaabaqadmaaaeaacqWGMbGzdaWgaaWcbaGaeGymaedabeaaaOqaaiabbAgaMjabb+gaVjabbkhaYbqaaiabbcdaWiabgsMiJIqaciab=fgaHjab=Xda8Gqaaiab+fdaXaqaaiabdAgaMnaaBaaaleaacqaIYaGmaeqaaaGcbaGaeeOzayMaee4Ba8MaeeOCaihabaGaeGymaeJaeyizImQae8xyaeMae8hpaWJae4NmaidabaGaemOzay2aaSbaaSqaaiabiodaZaqabaaakeaacqqGMbGzcqqGVbWBcqqGYbGCaeaacqqGYaGmcqGHKjYOcqWFHbqycqWF8aapcqGF5aqoaaaacaGL7baaaaa@5899@

where the maximum value taken by *f*_*1 *_*f*_*2 *_or *f*_*3 *_is equal to 1. Standard theory on point processes [24], gives the log-likelihood of the observed age-of-case data to be:

∑iNcaseslog⁡(πGi(ai,ti))−∑G∫∫πG(a,t)dadt
 MathType@MTEF@5@5@+=feaafiart1ev1aaatCvAUfKttLearuWrP9MDH5MBPbIqV92AaeXatLxBI9gBaebbnrfifHhDYfgasaacH8akY=wiFfYdH8Gipec8Eeeu0xXdbba9frFj0=OqFfea0dXdd9vqai=hGuQ8kuc9pgc9s8qqaq=dirpe0xb9q8qiLsFr0=vr0=vr0dc8meaabaqaciaacaGaaeqabaqabeGadaaakeaadaaeWbqaaiGbcYgaSjabc+gaVjabcEgaNjabcIcaOiabec8aWnaaBaaaleaacqWGhbWrdaWgaaadbaGaemyAaKgabeaaaSqabaGccqGGOaakcqWGHbqydaWgaaWcbaGaemyAaKgabeaakiabcYcaSiabdsha0naaBaaaleaacqWGPbqAaeqaaOGaeiykaKIaeiykaKIaeyOeI0YaaabuaeaadaWdbaqaamaapeaabaGaeqiWda3aaSbaaSqaaiabdEeahbqabaaabeqab0Gaey4kIipaaSqabeqaniabgUIiYdaaleaacqWGhbWraeqaniabggHiLdaaleaacqWGPbqAaeaacqWGobGtdaWgaaadbaGaem4yamMaemyyaeMaem4CamNaemyzauMaem4Camhabeaaa0GaeyyeIuoakiabcIcaOiabdggaHjabcYcaSiabdsha0jabcMcaPiabdsgaKjabdggaHjabdsgaKjabdsha0baa@610B@

The subscript *i *denotes actual case data; deaths are known to occur at age a_*i *_and a time t_*i *_after the start of the outbreak. Maximum likelihood methods were used to estimate the constant of proportionality, which determines the magnitude of the per capita rate of infection and the age-dependent susceptibility function as defined by *f*_*1*_, *f*_*2 *_and *f*_*3*_. We did this for (i) the 34 cases over the 10 year period assuming no differences between genotypes, and (ii) for the 28 genotyped cases allowing the 8 VRQ/ARQ cases to have either a lower susceptibility to infection or (iii) a longer incubation period than the 20 VRQ/VRQ cases. We found that models (ii) and (iii) produced a significant improvement in fit at the 95% level over model (i), but that the shape of the age-dependent susceptibility function was robust to the choice of model. Results are shown for model (ii).

For cattle, estimates of risk of BSE infection were made from n = 158,550 BSE cases in British cattle and were calculated from the cumulative distribution function, defined by Ferguson et al. [5], corresponding to the age-exposure/susceptibility curve (fitted using maximum likelihood methods).

For humans, estimates of risk of vCJD infection were obtained from a previous study that comprised n = 129 vCJD cases in British people, and were fitted using maximum likelihood methods by Boëlle et al. [6].

### Concordance between susceptibility data and anatomical data

For each combination of anatomical data and risk of infection estimates we calculated the Spearman's rank correlation coefficient, r_s_, between the value of the available measure of PP development (area, weight or number) and the risk of infection for an individual of the corresponding age. Sample sizes were *n *= 19, *n *= 94 and *n *= 46 for sheep, cattle and humans, respectively. Correlation coefficients were calculated using S-PLUS 2000 for Windows.

## Results

For sheep, cattle and humans alike, a strong correlation was found between risk of TSE infection and the development of lymphoid tissue in the gut which can explain both the relationships between age and disease incidence within species and differences in this relationship between species.

**Figure 1:**
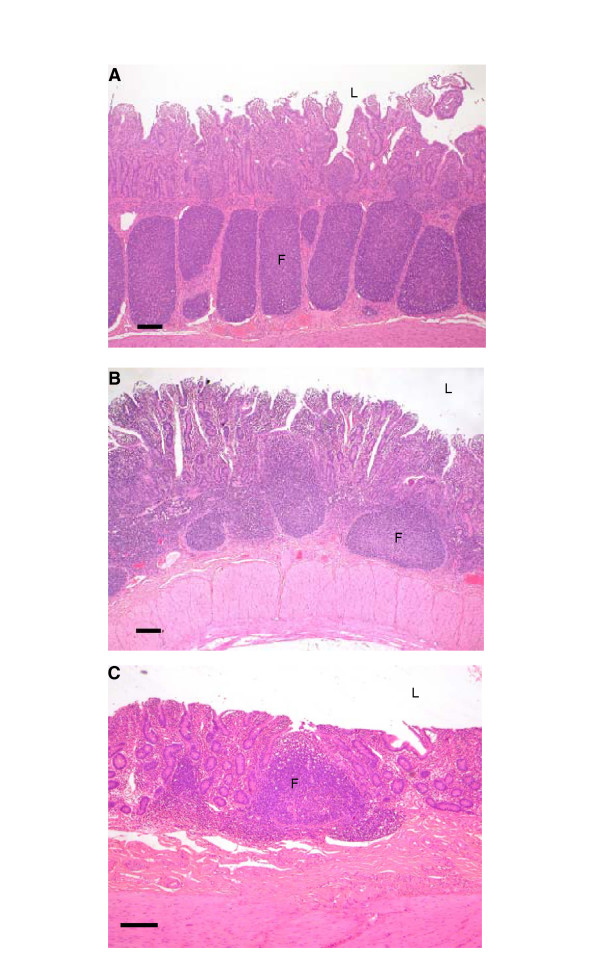
Comparison of Peyer's patch lymphoid follicles in the ileum of NPU Cheviot sheep at (A) 4 months, (B) 15 months, and (C) 6 years, using haematoxylin and eosin staining. F, lymphoid follicles undergo involution and are fewer in number with increasing age; L, intestinal lumen. Bar = 200 μm.

**Figure 2:**
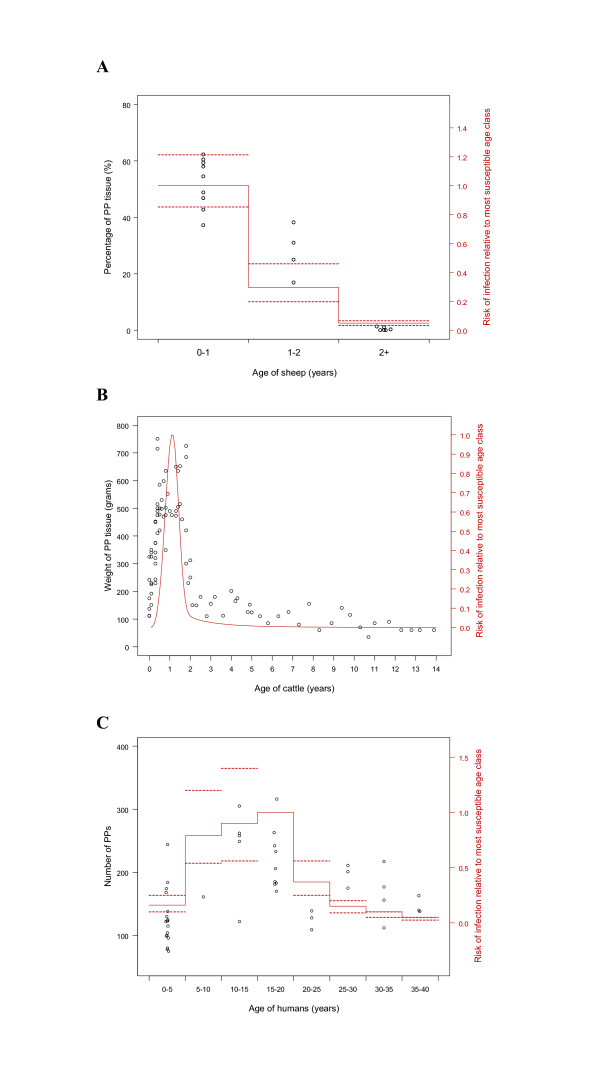
Comparison of age-related changes in Peyer's patch (PP) development and estimated risk of TSE infection relative to the most susceptible age class, for sheep, cattle and humans. (A) PP areas for n = 19 Cheviot sheep of mixed genotypes in 3 age classes (left hand axis, open circles), compared with estimates of risk of scrapie infection relative to the most susceptible age class (solid line) (± 50 percentiles-dashed lines) from field data on n = 34 cases in mixed PrP genotype (VRQ/VRQ and VRQ/ARQ) Cheviot sheep (see Methods) in the same age classes (right hand axis). (B) PP tissue weight against age for n = 94 cattle (open points, data from ref. 18), compared with estimates of risk of BSE infection relative to the most susceptible age class (solid line) as a function of age made from n = 158,550 BSE cases in British cattle [5] (C) Numbers of PPs in the small intestine in 8 age classes of humans (open circles, data taken from ref. 19), compared with estimates of risk of vCJD infection relative to the most susceptible age class (solid line, ± 50 percentiles-dashed lines) from n = 129 vCJD cases in British people for the same age classes (redrawn from ref. 6).

For sheep there is a marked fall in both the surface area of ileal PP tissue and lymphoid follicle density between approximately 12 and 24 months old, and both measures remain very low throughout adulthood (Figure 1 and 2(A)). Analysis of data on the incidence of natural scrapie over a 10 year period in the sheep flock providing the anatomical data indicates that the risk of infection is highest in the first year of life and is lowest in sheep >2 years old (Figure 2(A)). The two distributions peak in the same age class and are highly concordant (see Methods): surface area of ileal PP tissue vs risk of infection, r_s _= 0.913 (*n *= 19, P < 0.001); lymphoid follicle density vs risk of infection, r_s _= 0.933 (*n *= 19, P < 0.001).

For cattle, previous work [18] has shown that the weight of PP tissue in the small intestine increases in the first year of life, peaks at 12–18 months old, declines thereafter, and is low throughout adulthood (Figure 2(B)). Available estimates of age-related risk of infection of the British cattle population with BSE up to 1996 (given a mean incubation period of 5 years; published estimates range from 4.5 to 5.5 years [25]) indicate that the risk is initially low, peaks at about 12 months, and declines rapidly thereafter (Figure 2(B)). Again, the two distributions peak at similar ages and are concordant: r_s _= 0.693 (*n *= 94, P < 0.001).

For humans, previous work [19] has shown that the number of PPs in the small intestine increases during childhood, peaks at 10–15 years old, and declines thereafter, although the PPs persist throughout adulthood (Figure 2(C)). Recent estimates of age-related risk of infection of the British human population to vCJD [6] indicate that the risk is initially low, peaks between 5 and 20 years, and declines thereafter (Figure 2(C)). Here too, the two age-related patterns are concordant: r_s _= 0.38 (*n *= 46, P = 0.008). The same study [6] provides estimates of age-related susceptibility having allowed for changes in putative exposure associated with consumption of bovine carcass meat (see below). This is also concordant with the number of PPs: r_s _= 0.360 (*n *= 46, P = 0.014). Importantly, these correlations occur despite the markedly different patterns of age-related development of GALT in humans as compared with sheep and cattle.

## Discussion

Our results show that, whilst both age-related changes in the development of PP tissue and estimated risks of TSE infection differ between sheep, cattle and humans, in each case the two are associated. However, these results do not distinguish effects of age-related changes in exposure to TSE infection from age-related changes in susceptibility. To make this distinction we need to consider how oral exposure to TSE infection might change with age for each species.

For BSE in cattle, epidemiological studies have implicated meat and bone meal (MBM) containing recycled infected cattle tissues [26]. MBM used to be incorporated as a protein source in concentrated feedstuffs and fed to both calves and adult cattle. However, there is no clear correlation with the estimated age-infection function (Figure 2(B)): almost all calves were exposed to MBM by 6 weeks of age; exposure then fluctuated up to 24 months old but, especially for dairy cows, rose again in adulthood [27,28]. This route of BSE transmission is thought now to have been eliminated by feed production regulations introduced in 1988 and 1996.

For vCJD in humans, the most likely vehicle for exposure is food products containing BSE-contaminated cattle tissues [29]. Humans consume solid foods from 4–6 months of age with average consumption of bovine carcass meat peaking during childhood and tending to fall thereafter (see Figure 3 in [6]). This route of transmission is thought now to have been eliminated by food production regulations introduced in the UK in 1996. Here, putative exposure is more closely aligned with PP development [6] but, as reported above, when age-related exposure is taken into account, there remains an association between PP development and estimated susceptibility.

For scrapie in sheep, the vehicle(s) of oral exposure are less well understood, but are likely to include grazing on pasture contaminated with scrapie, possibly by infected foetal membranes [30]. Lambs typically begin to graze at 6–14 weeks and continue to do so throughout their lives. Exposure by this route would not be correlated with the estimated age-infection function (Figure 2(A)).

The importance of other transmission routes is less clear. Transmission from mother to offspring in utero or via breast milk (self-evidently age-dependent) is thought to play a minor role, if any: currently available estimates of the fraction of cases due to maternal transmission are 0–8% for scrapie in sheep [23], 0–14% for BSE in cattle [31], and 0% for vCJD in humans (Will et al., unpublished data). Other suggested routes include skin scarification (as demonstrated experimentally in mice [32]), food-borne infection via oral lesions [33], for scrapie possibly even mechanical transmission involving arthropods [34], and for vCJD, iatrogenic transmission [3]. However, there is no evidence that exposure via any of these routes varies with age in a manner corresponding to the estimated risk of infection functions (Figure 2)

The measures of PP development (area, weight or number) used in this study are crude indicators of lymphoid tissue development; alternative measures in PP development may be at least as appropriate (for example, in sheep, counts of functionally mature FDCs). Moreover, this analysis assumes that both the anatomical data and the age-susceptibility estimates available are representative of each host species in general and not just the specific populations examined. Similarly, it is assumed that the associations studied have not been distorted by other factors (e.g. history of exposure to gut pathogens) which might influence PP development and/or susceptibility to TSEs.

Given these caveats, it is nonetheless striking that an association between PP development and susceptibility to TSEs is seen not just in one host species but in three host species with different relationships between these variables and age. This kind of comparative study is especially useful in cases such as this where experimental manipulations (e.g. of PP development) are not feasible.

## Conclusion

Taken together, the epidemiological, anatomical and pathological evidence are consistent with the hypothesis that PP development or a close correlate of PP development is a major determinant of the observed age distribution of natural cases of TSEs in sheep, cattle and humans. This implies that the age groups most at risk of TSE infection (given that the individuals are exposed and have a susceptible PrP genotype) are indicated by the development of Peyer's patches in the gut.

## Competing interests

The author(s) declare that they have no competing interests.

## Authors' contributions

SGS participated in all aspects of the study, anatomical studies of sheep and preparation of the manuscript. NH collected the sheep data, provided advice on database information and participated in the interpretation of findings. LM participated in the data analysis and interpretation of findings. JDF participated in the collection of data and interpretation of findings. MECT participated in the analysis, interpretation and presentation of findings. LEBK participated in the data analysis and interpretation of findings. DJS participated in the analysis, interpretation and presentation of findings. SMR participated in the acquisition and interpretation of gross and anatomical data. RGW participated in the interpretation of findings and preparation of the manuscript. MEJW participated in the interpretation of findings and preparation of the manuscript. All authors read and approved the final manuscript.

## Pre-publication history

The pre-publication history for this paper can be accessed here:


